# Estrogen and Progesterone Regulate p27kip1 Levels via the Ubiquitin-Proteasome System: Pathogenic and Therapeutic Implications for Endometrial Cancer

**DOI:** 10.1371/journal.pone.0046072

**Published:** 2012-09-27

**Authors:** Kuang-Tzu Huang, Savvas C. Pavlides, Jon Lecanda, Stephanie V. Blank, Khushbakhat R. Mittal, Leslie I. Gold

**Affiliations:** 1 Department of Medicine, New York University School of Medicine, New York, New York, United States of America; 2 Department of Pathology, New York University School of Medicine, New York, New York, United States of America; 3 Department of Obstetrics and Gynecology, New York University School of Medicine, New York, New York, United States of America; 4 New York University Cancer Institute, New York, New York, United States of America; Florida International University, United States of America

## Abstract

The levels of proteins that control the cell cycle are regulated by ubiquitin-mediated degradation via the ubiquitin-proteasome system (UPS) by substrate-specific E3 ubiquitin ligases. The cyclin-dependent kinase inhibitor, p27kip1 (p27), that blocks the cell cycle in G1, is ubiquitylated by the E3 ligase SCF-Skp2/Cks1 for degradation by the UPS. In turn, Skp2 and Cks1 are ubiquitylated by the E3 ligase complex APC/Cdh1 for destruction thereby maintaining abundant levels of nuclear p27. We previously showed that perpetual proteasomal degradation of p27 is an early event in Type I endometrial carcinogenesis (ECA), an estrogen (E2)-induced cancer. The present studies demonstrate that E2 stimulates growth of ECA cell lines and normal primary endometrial epithelial cells (EECs) and induces MAPK-ERK1/2-dependent phosphorylation of p27 on Thr187, a prerequisite for p27 ubiquitylation by nuclear SCF-Skp2/Cks1 and subsequent degradation. In addition, E2 decreases the E3 ligase [APC]Cdh1 leaving Skp2 and Cks1 intact to cause p27 degradation. Furthermore, knocking-down Skp2 prevents E2-induced p27 degradation and growth stimulation suggesting that the pathogenesis of E2-induced ECA is dependent on Skp2-mediated degradation of p27. Conversely, progesterone (Pg) as an inhibitor of endometrial proliferation increases nuclear p27 and Cdh1 in primary EECs and ECA cells. Pg, also increases Cdh1 binding to APC to form the active E3ligase. Knocking-down Cdh1 obviates Pg-induced stabilization of p27 and growth inhibition. Notably, neither E2 nor Pg affected transcription of Cdh1, Skp2, Cks1 nor p27. These studies provide new insights into hormone regulation of cell proliferation through the UPS. The data implicates that preventing nuclear p27 degradation by blocking Skp2/Cks1-mediated degradation of p27 or increasing Cdh1 to mediate degradation of Skp2-Cks1 are potential strategies for the prevention and treatment of ECA.

## Introduction

Estrogen (E2) stimulates proliferation of the endometrium and progesterone (Pg) suppresses E2-driven proliferation. Aligned with the effects of these hormones on growth, E2 induces type I endometrial carcinoma (ECA; rate: 85% of all ECAs) and conversely, Pg is used as a therapeutic agent for endometrial hyperplasia, the precursor to ECA [1]. ECA is the most common gynecological malignancy with an incidence of 136,000 global cases per year [2]. At least 50% of women with endometrial atypical hyperplasia (AEH) have concurrent ECA; an additional 30% will progress to ECA [3]. As an alternative to hysterectomy, progestins reverse AEH and well-differentiated ECA leading to a high rate of successful pregnancies [4], [5]. A molecular level understanding of normal and malignant growth regulation of the endometrium by E2 and Pg is important to advance the field in terms of defining novel preventative and therapeutic molecular targets for this disease. We previously reported that the cyclin-dependent kinase (Cdk) inhibitor, p27kip1 (p27) critical to growth arrest, is absent in the glands of both AEH and ECA tissue due to rapid and perpetual degradation of p27 via the ubiquitin proteasome system (UPS) implicating loss of p27 occurs early in the oncogenesis of ECA [6]. Aligned with the opposing effects of E2 and Pg on proliferation, we further showed that E2 caused proteasomal degradation of p27 in primary EECs whereas Pg markedly increased p27 in both primary endometrial epithelial cells (EECs) and ECA cells. These data suggest that p27 is a significant molecular target involved in both the pathogenesis and treatment of ECA.

As a tumor suppressor and member of the Cip/Kip family of Cdk inhibitors, p27 arrests cell proliferation in G1 phase of the cell cycle by blocking cyclinE/Cdk2 activity [7]. Unlike other tumor suppressors and negative regulators of the cell cycle, the p27 gene *CDKN1B*, is rarely mutated in human cancers but instead p27 levels are highly regulated by post-translational modifications [7]. The concentration, subcellular localization, and phosphorylation of specific amino acids within the p27 molecule ultimately regulate its effect on Cdk2 activity and thus, cell proliferation. p27 translation and protein stability are highest in G0 and early G1 when p27 binds to and inhibits CyclinE/Cdk2 for cell cycle arrest. As the cell cycle progresses through G1, incremental decreases in p27 increase the activity of CylcinE and CyclinA bound to Cdk2, which activate genes involved in progression from G1 to S and the initiation of DNA replication [8]. At lower concentrations, p27 is phosphorylated on T187 [p-p27(T187)] by CyclinE/Cdk2, which retains p-p27(T187) in the nucleus where it is ubiquitylated, which is required for degradation by Skp2/Cks1 of the SCF^Skp2^ E3 ligase complex [7], [9]. Phosphorylation of p27 on T187 requires previous tyrosine phosphorylation of p27 at three sites in its Cdk2 inhibitory domain, which can be achieved by Abl or Src family kinases [10]. Phosphorylation of p27 on other amino acid residues by different kinases dictates its subcellular localization [7], [11], [12], [13], [14]. Whether p27 is degraded in the nucleus or sequestered in the cytoplasm, cell cycle arrest will not occur unless there are sufficient levels of nuclear p27 to inhibit Cdk2. Importantly, in the nucleus, p27 is tumor suppressive but in the cytoplasm, it is oncogenic as it medicates migration/metastasis [15], [16], [17]. As in ECA, lack of nuclear p27 has been found in numerous epithelial malignancies, [6], [7], [17], [18], [19] and both an inverse relationship between the levels of nuclear p27 (low) and Skp2 (high) and sequestration of p27 in the cytoplasm is associated with decreased survival [7], [15], [20], [21].

The SCF (Skp1-Cul1-Skp2/Fbox; SCF-Skp2/Cks1) and anaphase promoting complex (APC; APC/Cdh1; APC/Cdc20) are multi-subunit E3 ligase complexes of the UPS that cause proteasomal degradation of Cyclin/Cdks and their Cdk inhibitors with precise timing to synchronize and regulate progression and arrest of the cell cycle in a cyclical manner [22], [23]. Polyubiquitin conjugation of protein substrates on lysines is achieved by three enzymes, which transfers/activates (E1), conjugates (E2) and ligates (E3) ubiquitin to the protein for selective recognition and degradation by the 26S proteasome [24]. The F-box protein, Skp2, is the substrate recognition module of SCF-Skp2/Cks1. Skp2 interfaces with the accessory protein Cks1 forming a pocket that identifies p-p27(T187) and ubiquitylates the molecule on lysines 134, 153, and 165 [22], [23], [25]; Skp2 and Cks1 are rate limiting for p27 degradation [9]. In late G1-S when the level of SCF-Skp2/Cks1 is highest, Skp2 and Cks1 become substrate recognition targets for APC/Cdh1 E3 ligase complex ubiquitin-mediated degradation [26]. This degradation of Skp2 and Cks1 results in nuclear accumulation of p27 for G1 arrest [7], [9], [27]. Taken together, an increase in APC/Cdh1 indirectly increases nuclear p27 levels by causing degradation of Skp2 and Cks1 and this decrease in Skp2/Cks1 directly increases nuclear levels of p27. Furthermore, the levels of nuclear Skp2 and p27 are inversely correlated with growth stimulation and growth inhibition in the context of being prooncogenic or tumor suppressive, respectively.

The present studies show a novel mechanism by which ovarian steroids regulate the growth of the endometrium, which is through manipulation of the levels of proteins of the UPS and not by transcription. Specifically, we report that E2 causes MAPK-ERK1/2-dependent phosphorylation of nuclear p27 on T187 and decreases the E3 ligase APC/Cdh1 leaving Skp2/Cks1 intact to ubiquitylate nuclear p27 for degradation resulting in stimulation of proliferation of EECs and ECA cell lines. Conversely, Pg increases Cdh1 and its binding to APC; the APC/Cdh1 E3 ligase causes proteasomal degradation of Skp2 and Cks1, leaving p27 intact for growth inhibition. We propose that p27 is a significant molecular target in hormone-driven growth control of the endometrium and that preventing p27 degradation by either blocking Skp2/Cks1 or increasing Cdh1 are rational approaches for therapeutic intervention of AEH and ECA.

## Results

### Estrogen and progesterone have opposite effects on cell proliferation and the levels of p27, Skp2, and Cks1 proteins

**Figure 1:**
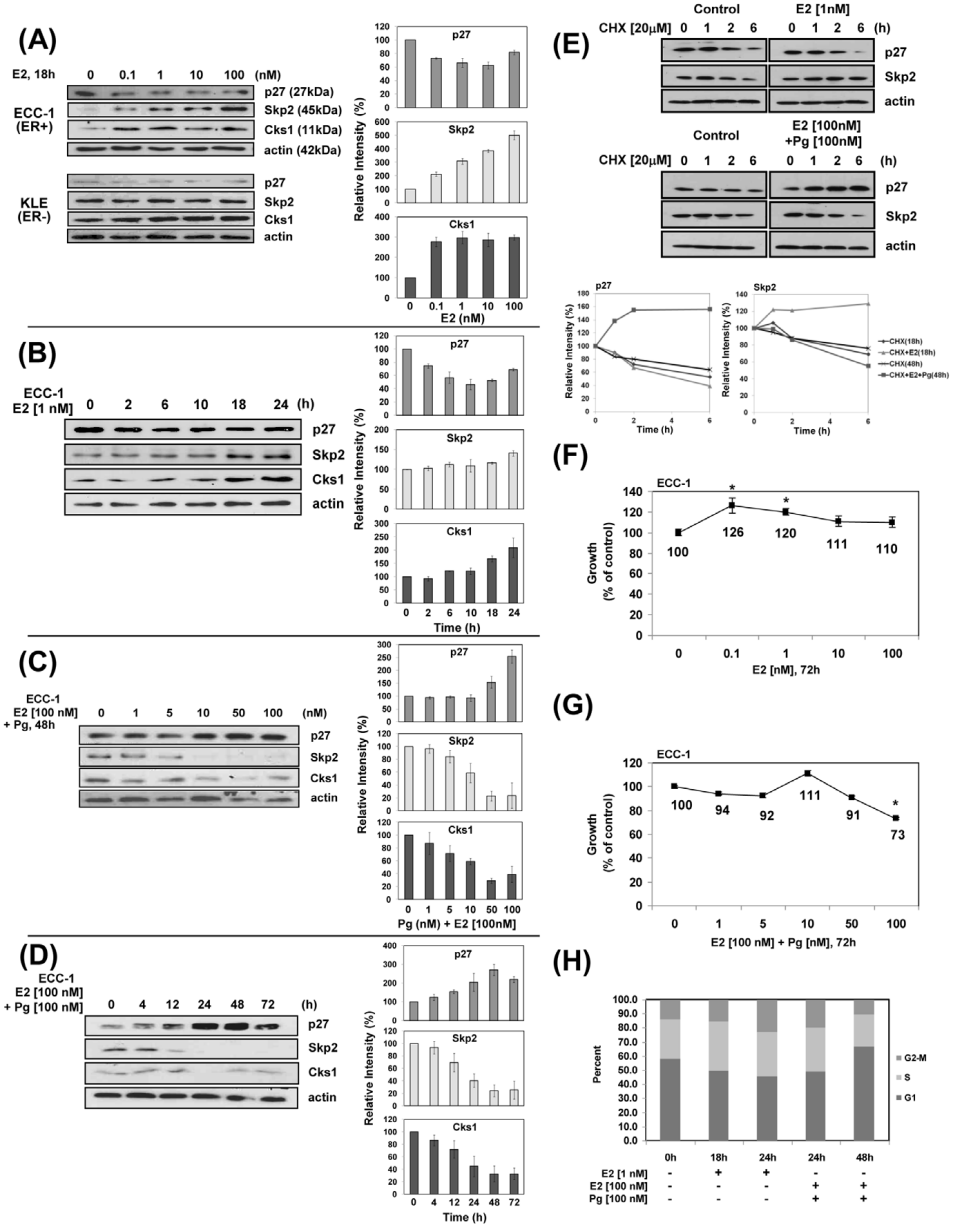
Estrogen (E2) and progesterone (Pg) have opposite effects on cell proliferation and levels of p27, Skp2, and Cks1 proteins. Details are in Materials and Methods and each Figure. A, E2 dose-response: ECC-1 and KLE cells were treated with E2 and p27, Skp2, and Cks1 protein levels determined by immunoblotting. B, E2 time course: ECC-1 cells were treated with E2 for the times indicated and protein levels determined as above. C, Pg dose-response: ECC-1 cells were treated with Pg/E2 and p27, Skp2 and Cks1 protein levels determined as above. D, Pg time course: ECC-1 cells were treated with Pg/E2 for the times shown and protein levels as above. Densitometric scans of all protein bands are shown by the graphs to the right of each blot. Each band was normalized to actin and then compared with 0 time or untreated control. The data are expressed as relative intensity of each band ± standard deviation. E, E2/Pg cycloheximide (CHX) treatment: ECC-1 cells were treated with E2 for 18 h or with E2 and Pg for 48 h. CHX was added 6 h prior to final harvest. Cells were harvested at 0,1,2, and 6 h time points. CHX alone was the baseline control. Lysates were prepared and immunoblotted for p27 and Skp2 and the levels of each protein band determined by densitometry (band intensity). The percent change within each treatment parameter was calculated based on the zero time point for each group. Protein turn-over was determined by comparison of cells treated with CHX alone compared to CHX in the presence of each hormone at each time point. The line graphs represent the relative intensity of each band normalized to actin for each group. F, G: E2 stimulates and Pg inhibits proliferation: ECC-1 cells were treated with E2 or Pg/E2 and cell proliferation determined by MTS assay (Materials and Methods) *p<0.05. H, Cell cycle distribution: ECC-1 cells were treated with E2 or E2/Pg and cell cycle analysis performed as described Materials and Methods.

Consistent with hormonal growth regulatory effects on the endometrium, we previously showed that the ovarian hormones, estrogen (E2) and progesterone (Pg; in the presence of E2 to increase Pg receptors [PR] for a physiological response that mimics the menstrual cycle [6], [28], [29]) had opposite effects on the levels of p27 in primary EECs such that E2 decreased the levels of p27 via ubiquitin-mediated degradation and conversely, Pg induced a marked increase in p27 levels in both EECs and primary ECA cells [6]. Since Skp2 and Cks1, as components of the SCF^Skp2^ E3 ligase cause degradation of p27 [7], [9], [23], we proposed that E2 and Pg would similarly inversely effect the levels of Skp2 and Cks1 for stimulation and inhibition of cell proliferation, respectively. Using ECC-1 cells expressing both ER and PR, we show that E2 induced a dose-dependent and time dependent decrease in p27 by 36% and 33% with peak responses of 1 and 10 nM, respectively, and inversely affected Skp2 and Cks1 levels with a 5.4-fold and 3-fold increase, respectively, with a peak response of 100 nM E2 (**Figure 1A, B**). The decrease in p27 occurred as early as 2 h and was maintained through 24 h whereas the increase in Skp2 and Cks1 occurred later, peaking at 24 h. The ER negative KLE cell line did not respond to E2 but instead, expressed high levels of Skp2 and Cks1 leaving little to no p27. The opposite effect was shown for Pg (plus E2), which induced a 2.8-fold increase in p27 at 100 nM Pg and a concomitant 84% and 75% decrease in Skp2 and Cks1, respectively (**Figure 1C**). p27 incrementally increased from 12–48 h with a 3-fold increase at 48 h that waned to 2-fold at 72 h whereas Skp2 and Cks1 decreased over time and were nearly absent by 24 h (**Figure 1D**). It is important to note, that the exposure times of the blots were less following induction by Pg compared to E2 since the levels of p27 were so abundant and could not have been quantified compared to actin. Thus, the levels of p27 at zero time for the blots showing E2-induced decreases in p27 were consistently higher and those showing the Pg-induced increases were consistently lower. To more quantitatively estimate the effects of E2 and separately, Pg (plus E2) on p27 and Skp2 stability, ECC-1 cells were incubated with cycloheximide (CHX) alone or in the presence of E2 or Pg. As shown in **Figure 1E**, after 6 h in culture, E2 decreased the levels of p27 by 61% compared to 47% by CHX alone yielding an estimated decrease in p27 half-life by 1.5 h (6.2 h versus 4.7 h; total incubation time = 18 h). Consistent with the more rapid degradation of p27 induced by E2, Skp2 protein levels were increased by 1.3 fold at this time point. In contrast, Pg stabilized p27 as the level of p27 protein was increased by 1.5 fold at 6 h while Skp2 protein levels were decreased by 45% compared to 24% by CHX alone yielding and estimated decrease in Skp2 half-life by 6.2 h (12.9 h versus 6.7 h total incubation time = 48 h).

Consistent with the peak concentrations of E2 and Pg effects on p27, Skp2, and Cks1, E2 induced cell proliferation with peak responses of 26–20% over untreated controls at 0.1 nM and 1 nM (**Figure 1F**) respectively, whereas Pg inhibited proliferation with a peak response of 27% with at 100 nM (**Figure 1G**). Following cell cycle analysis, we confirmed that the hormones affect cell proliferation as reflected by their effects on cell cycle distribution (**Figure 1H**). After 18 h treatment with E2, the number of ECC-1 cells in G1 decreased from 58% to 49% and increased in G2 and S from 14% to 15% and 29% to 36%, respectively. After Pg treatment at 48 h, the fraction of cells in G1 increased from 58% to 67% and decreased in G2 and S from 13% to 10% and 27% to 23%, respectively.

### Estrogen induced ERK-dependent phosphorylation of p27 on Thr187

**Figure 2:**
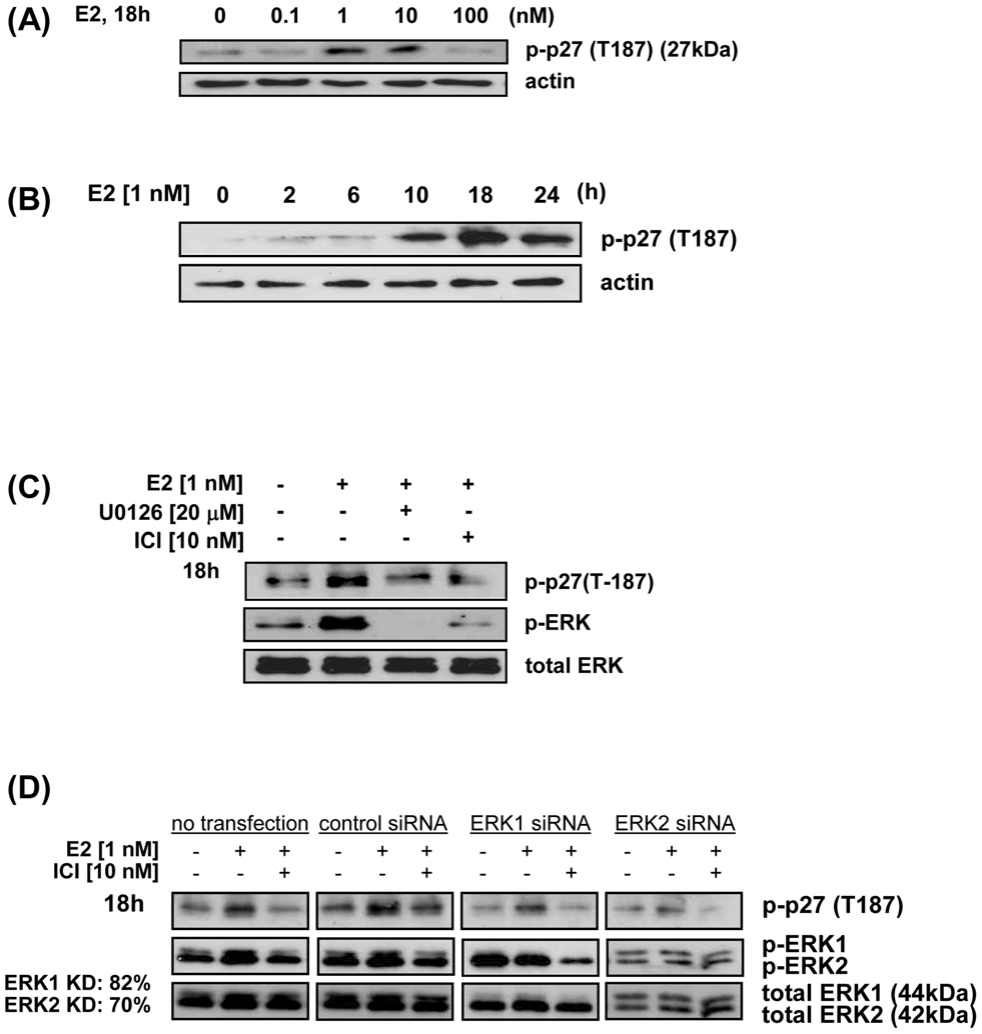
E2 induces ERK-dependent phosphorylation of p27 at Thr187. A, E2 dose-response: EEC-1 were treated with E2 and immunoblot analysis performed using rabbit anti-human phospho-p27 (p-p27[T187]), as described in Materials and Methods. B, E2 time course: ECC-1 cells were treated with E2 and protein levels determined by immunoblotting as in A. C, E2, phospho-ERK: ECC-1 cells were treated with E2 in the presence of U0126 or ICI and cell lysates immunoblotted for p-p27(T187), phospho ERK (p-ERK), and total ERK, as described in Materials and Methods. D, E2, Analysis of ERK1 and ERK2 activation following their knock-down with siRNA: ECC-1 cells were transfected with ERK1, ERK2, or control siRNA, treated with E2 in the presence or absence of ICI, followed by cell fractionation, and immunoblotting for total and phospho-ERK-1(p-ERK1) and ERK2 (p-ERK2), as described in Materials and Methods.

Phosphorylation of p27 at T187 confers the molecular conformation for its ubiquitylation by SCF-Skp2/Cks1 [22]. **Figure 2A** illustrates that E2 induced a 2.5-fold and 2.3-fold increase in phosphorylation of p27 at T187 (p-p27[T187]) at 1 nM and 10 nM E2 respectively in ECC-1 cells (and in HEC-1B cells; Figure S1A), with an 11-fold peak response of p-p27(T187) at 18 h post treatment (**Figure 2B**) that was MAPK-dependent (Figure S1B). As shown here, this is the identical peak dose and time response in which p27 is degraded by E2 (consistent with Figures 1A and 1C) in ECC-1 cells. In initial experiments, neither the proteasome inhibitor, lactacystin and the serine/threonine phosphatase inhibitor, okadaic acid were necessary to maintain p27 in the highly p-p27(T187) state suggesting that the rate of proteasomal degradation of p-p27(T187) is slower than phosphorylation of T187. We further show that ERK is activated by E2 downstream from MEK1 (blocked by U0126) (**Figure 2C**). From these data, we conclude that E2-induced phosphorylation of p27 at T187 are MAPK/ERK-dependent and ER-specific responses. Finally, siRNA knock-down of ERK1 and separately, ERK2 (82%, 70% efficiency, respectively) decreased p-p27(T187) in the untreated knockdown cells compared to control siRNA (**Figure 2D**). As shown in both the untransfected and control siRNA transfected cells, it appears that ERK2 is more highly expressed than ERK1 (n.b. this might be due to a higher specificity of the antibody for ERK2 than ERK1). The blot shows that knocking down ERK2 decreased the phosphorylation of p27 to a greater extent than knocking-down ERK1 (compare levels of p-p27(T187) in ERK1 and ERK2 knock-down to control siRNA). Because of the knock-down efficiency and the higher expression of ERKs by these cells with and without E2-induction, it is difficult to quantify the contribution of ERK1 and ERK2 in the E2-induced MAPK-driven phosphorylation of p27 at T187. Nonetheless, it appears that E2 activates both ERK1 and ERK2 for the phosphorylation of p27 on T187 for recognition by SCF-Skp2/Cks1 (**Figure 2D** and Figure S1B).

### Knocking down Skp2 increased nuclear and cytoplasmic p27 and blocked estrogen-induced degradation of p27 [by SCF-Skp2/Cks1] in both cellular compartments

**Figure 3:**
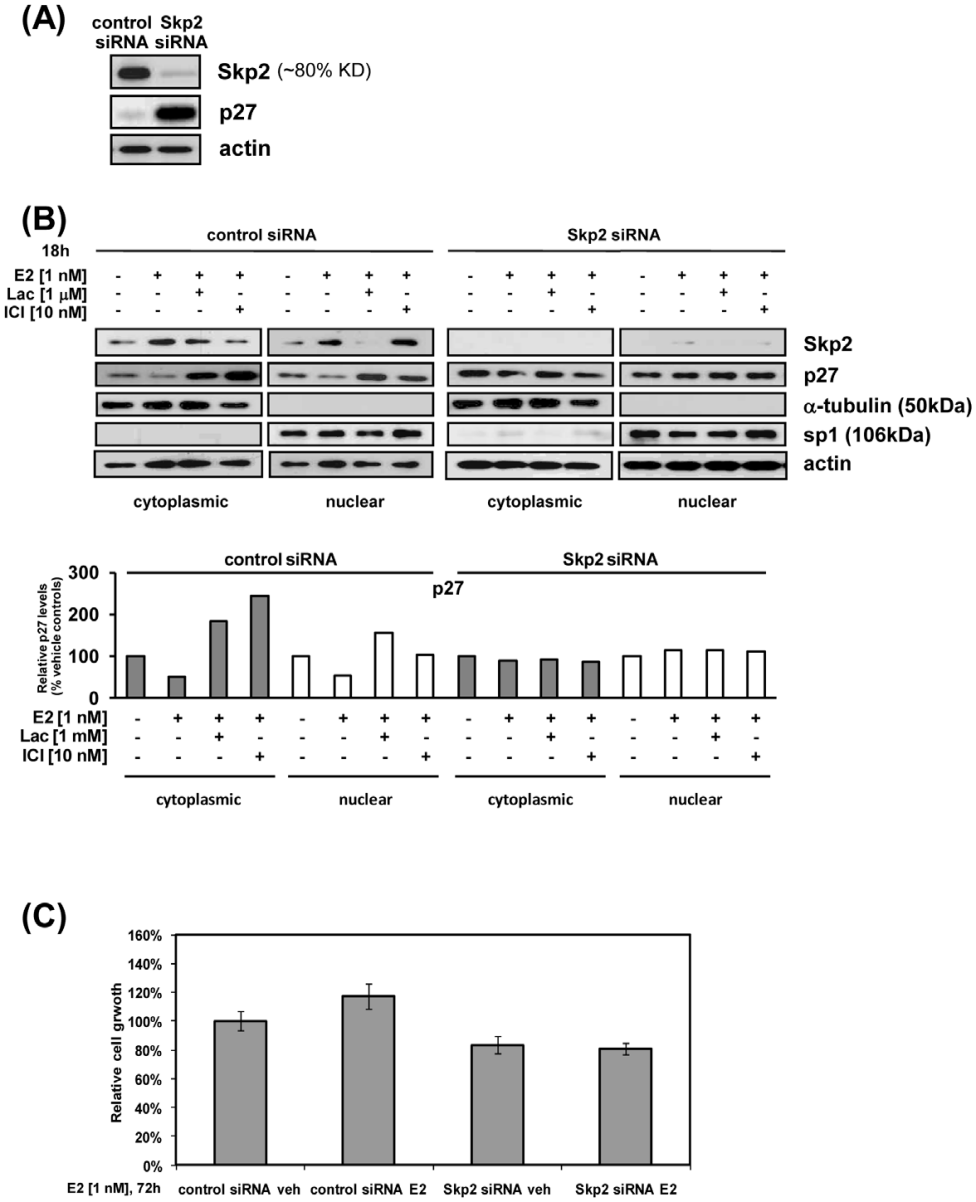
Knock-down of Skp2 blocks E2-induced UPS-mediated degradation of p27 in the nucleus and cytoplasm. A, B, E2, Skp2 knockdown: ECC-1 cells were transiently transfected with Skp2 or control siRNA, treated with E2 alone or in the presence of 1 µM Lactacystin (Lac) or 10 nM ICI, subjected to cell fractionation, p27 and Skp2 levels determined by immunoblotting, as described in Materials and Methods. In Panel A, knock-down efficiency was determined by comparing cell lysates from Skp2 siRNA and control siRNA treated cells by immunoblotting. Relative p27 levels are represented by the densitometric scan below the blot. E2, cell proliferation in Skp2 knockdown cells: ECC-1 cells, transfected with Skp2 or control siRNA, were treated with E2 or not treated and proliferation determined by MTS assay, as described in Materials and Methods. Data are presented as an average of two independent experiments. *p≤0.05.

We clearly show that as Skp2 and Cks1 levels are increased, p27 levels decrease with growth stimulation in response to E2 (Figures 1A,B,E). As shown in **Figure 3A**, knocking down Skp2 (SKP2 siRNA; 80% efficiency) caused an 8.2-fold increase in p27. The degradation of p27 by Skp2 is reported to occur in the nucleus [9], [30]. However, we found that E2 induced a 2.5 fold and 1.9 fold increase in Skp2 in the nucleus and cytoplasmic fractions, respectively and a 50% decrease in p27 in both fractions (**Figure 3B** **, left panels**). Importantly, we demonstrate that following knockdown of Skp2, E2-induced p27 degradation was blocked in the cytoplasm and nucleus (**Figure 3B** **, right panels** and Figure S2), that stimulation of cell proliferation by E2 was completely obviated (**Figure 3C**), and that the E2-induced decrease in p27 was ER- and proteasome-dependent in both subcellular fractions (**Figure 3B** **, left panel**). Likely, the decrease in growth following Skp2 knockdown is due to the increase in levels of p27 (as shown in **Figure 3B**, right panel). These experiments implicate Skp2 of the SCF-Skp2/Cks1 as a direct mediator of E2-induced degradation of p27.

### Estrogen and progesterone have opposite effects on Cdh1 protein levels to ultimately regulate the level of nuclear p27 protein; Pg and E2 do not affect mRNA levels of Cdh1, Skp2, Cks1 or p27

**Figure 4:**
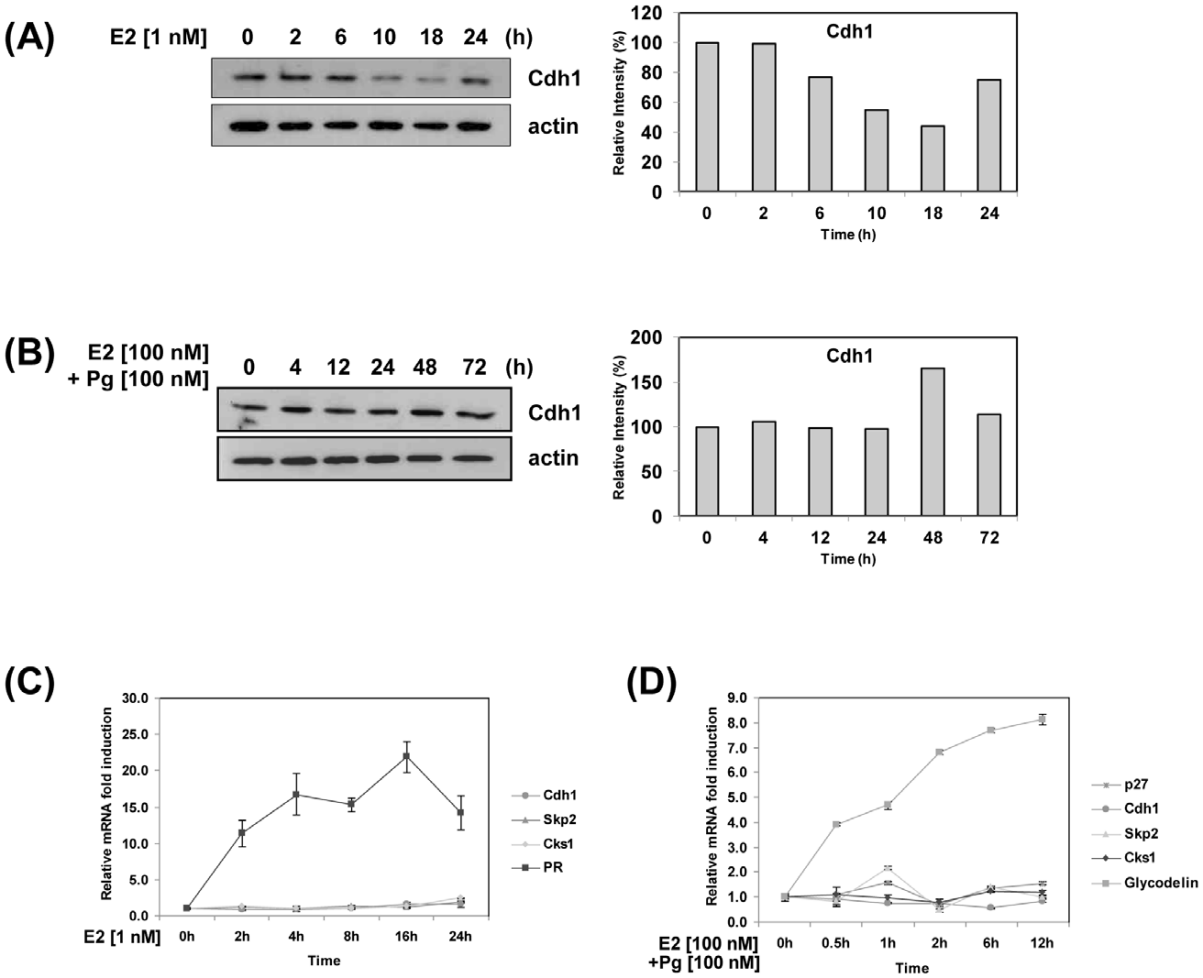
Time course: Opposite effect of E2 and Pg on Cdh1 protein levels; Pg and E2 do not affect mRNA levels of Cdh1, Skp2, Cks1 or p27. A, B, Time course of E2 and Pg [plus E2] treatment of EEC-1 cells. ECC-1 cells were treated with E2 and Pg plus E2, the experiments terminated at the times indicated, lysates prepared, and Cdh1 protein levels determined by immunoblotting, as described in Materials and Methods. C, D, E2, Pg, mRNA levels of p27, Skp2, Cks1 and Cdh1: ECC-1 cells were treated with E2 or Pg/E2, total RNA extracted at the times shown, and quantitative real-time RT-PCR performed, all in Materials and Methods. PR and glycodelin primers were used as controls for ER and PR target genes, respectively. Data are presented as an average of two independent experiments.

The ubiquitylation and degradation of Skp2 and Cks1 by the E3 ligase APC/Cdh1 would prevent the degradation of p27. Therefore, we hypothesized that [APC]Cdh1 levels would vary directly with p27 and therefore be regulated in an opposing manner by E2 and Pg. Consistent with this hypothesis, whereas E2 treatment of ECC-1 cells time-dependently decreased Cdh1 with a maximum effect of 56% at 18 h (**Figure 4A**), Pg-treatment increased Cdh1 over time with a peak response of 1.7-fold at 48 h (**Figure 4B**). In contrast neither E2 nor Pg affected transcription of Cdh1, p27, Skp2, or Cks1 at early time points through 24 h (**Figure 4C**), and 12 h (**Figure 4D**), respectively, whereas the mRNA response of common genes containing transcriptional responsive elements for ER and PR, namely, PR and glycodelin, respectively, is high (**Figure 4D**).

**Figure 5:**
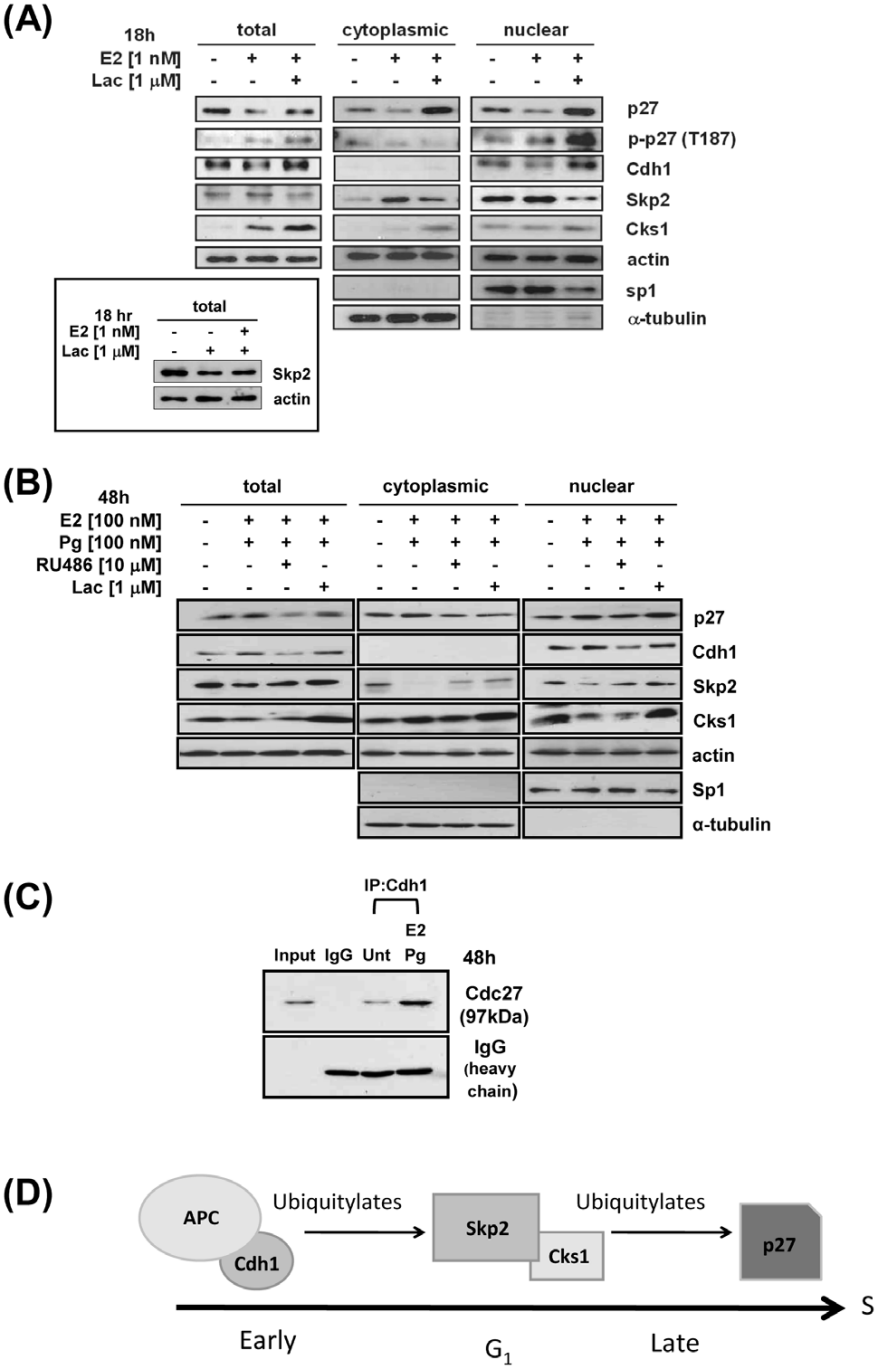
Subcellular fractionation: opposite Effects of E2 and Pg on Cdh1 protein levels compared to p27, p-p27(T187), Skp2, and Cks1; Pg induces binding of Cdh1 to APC. A, E2; B, Pg, Cell fractionation and analysis of Cdh1, Skp2, Cks1, p27 proteins and p-p27(T187): Cells were either untreated, treated with E2, E2 plus Lac or treated with Pg/E2 with or without 1 µM Lactacystin (Lac) or RU486. Cells were fractionated and proteins analyzed by immunoblotting, all described in Materials and Methods. The *inset in A* shows that lactacystin decreases Skp2 protein levels. C., Pg, Cdh1 binding to APC: Cells were treated with Pg/E2, cell lysates immunoprecipitated with anti-Cdh1 followed by immunoblotting with anti-Cdc27, as described in Materials and Methods. D, diagram depicts APC/Cdh1 as the upstream E3 ligase that ubiquitylates Skp2/Cks1 (of the SCF complex), which is the downstream E3 ligase that ubiquitylates p27 to regulate p27 protein levels.

Following E2- treatment of ECC-1 cells and subsequent subcellular fractionation, a 21% decrease in Cdh1 was shown in the nuclear fraction. This decrease in the level of Cdh1 was reversed by lactacystin (**Figure 5A**) suggesting that E2 regulates the levels of Cdh1 by proteasomal degradation. Interestingly, the level of Skp2 was decreased by 18% with E2 treatment in the presence of lactacystin in both the nucleus and cytoplasm and also, by lactacystin alone, shown here in total cell lysates (by 37%; inset of Figure 5A). This result is difficult to explain and although lactacystin is widely used to inhibit proteasome degradation, there might be off-target effects, such proteolysis of Skp2 by different degradation pathways.

It is notable that unlike Skp2, Cks1, and p27, Cdh1 is not present in the cytoplasm. A concurrent decrease in p27 was observed in both the nuclear and cytoplasmic fractions by 30% and 61%, respectively, compared to untreated controls (basal p27 was higher in the nucleus than the cytoplasm of untreated cells). Consistent with the nuclear decrease in p27, p-p27(T187) was increased for the identification and ubiquitylation of p27 by Skp2/Cks1 for its degradation. Moreover, lactacystin inhibited p27 degradation in both the nucleus and cytoplasm and increased p-p27(T187) by 2.4-fold only in the nuclear fraction confirming that p27 is phosphorylated on T187 in the nucleus and furthermore, remains and accumulates in the nucleus when its degradation is inhibited by lactacystin (same as Figure 3B, left panels). An anticipated inverse relationship between p27 and Skp2 levels was shown following E2-treatment in both the nuclear and cytoplasmic fractions. Conversely, Pg treatment of ECC-1 cells induced a 1.65-fold increase in both Cdh1 and p27 in the nucleus while this hormone decreased Skp2 by 66% in the cytoplasm and both Skp2 and Cks1 by 62% in the nuclear fraction; these responses were PR-specific (**Figure 5B**). Unlike E2 treatment in which p27 degradation occurred in both cellular compartments, p27 was largely stabilized in the nucleus by Pg (1.7 versus 1.1-fold). It is possible that Skp2 and Cks1 were degraded via the UPS since lactacystin increased Skp2 and Cks1 in the nucleus and Cks1 in the cytoplasm as well. Whereas we show Cdh1 levels are increased by Pg, this protein must be bound to the APC complex [in a hypophosphorylated state] to exert its specific E3 ligase activity [31], [32], [33]. As shown in **Figure 5C**, Pg indeed increased the binding of Cdh1 to APC ostensibly increasing its E3 ligase activity for Skp2 and Cks1 and thus, their proteasomal degradation. Taken together, as shown in **Figure 5D**, our results implicate Cdh1 as the upstream regulator of the levels of p27 through the UPS (i.e., SCF-Skp2/Cks1) and the critical link to effectuate hormonal regulation of growth.

### Knocking down Cdh1 blocks Pg-mediated accumulation of nuclear p27 and growth inhibition

**Figure 6:**
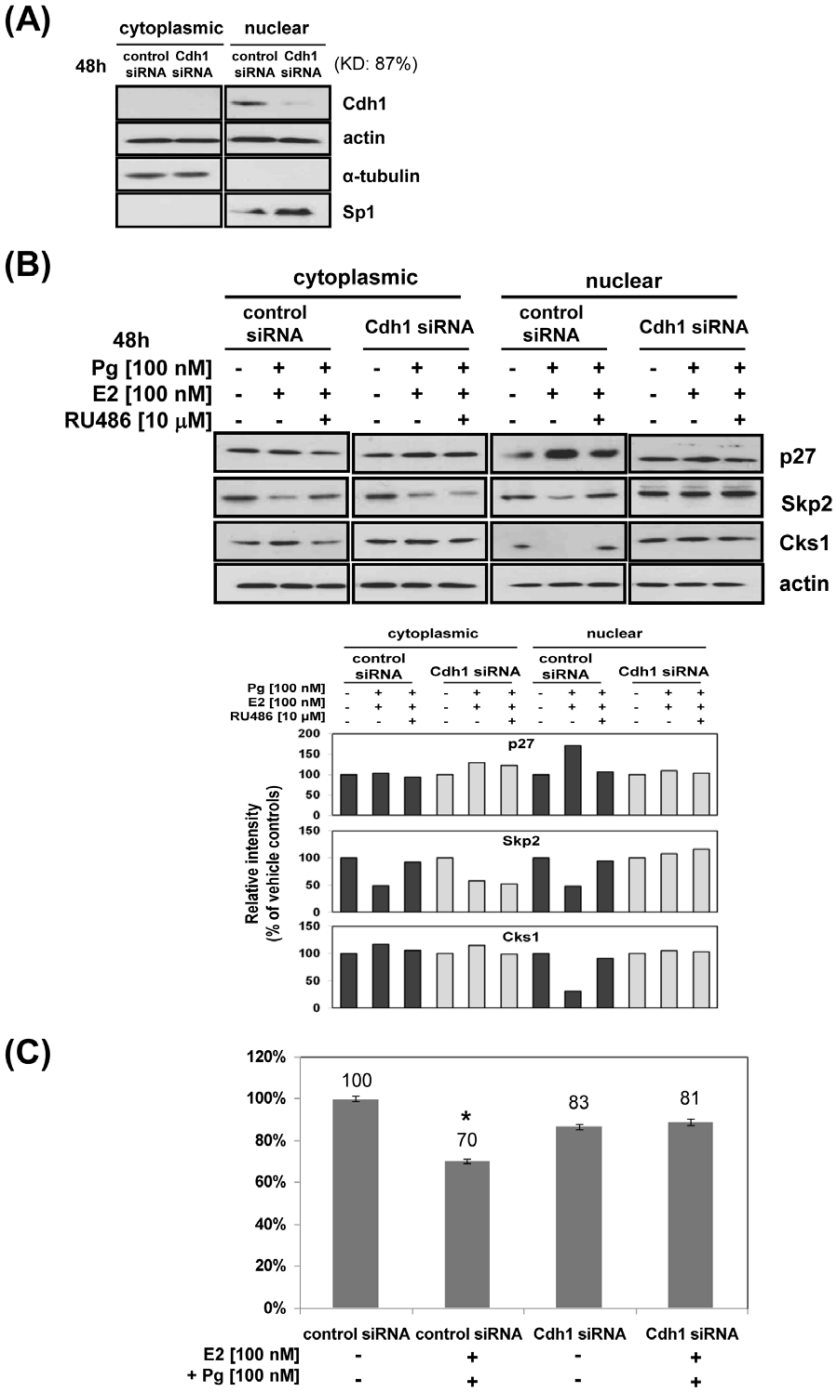
Knock-down of Cdh1 blocks Pg-mediated increase in nuclear p27 and growth inhibition. A, Pg, Cdh1 knockdown: ECC-1 cells were transiently transfected with Cdh1 siRNA followed by cytoplasmic/nuclear separation, and knockdown efficiency determined in each fraction by comparing control siRNA and Cdh1 siRNA transfected cell lysates. B, Pg, Cdh1 knock-down and subcellular fractionation: transfected cells were treated with Pg/E2 with or without RU486, subjected to cellular fractionation, and immunoblot analysis performed on knock-down and control siRNA cells for proteins shown; all described in Materials and Methods. Densitometric scans (relative intensity) of each protein band representing response levels relative to actin and compared with untreated controls are shown in the graph below. C, Pg, cell proliferation in Cdh1 knockdown cells: cells transfected with Cdh1 or control siRNA were treated Pg/E2 or not treated and proliferation analyzed by MTS assay as described in Materials and Methods; *p<0.05.

**Figure 6A** illustrates that knocking down Cdh1 (*fzr* gene; 87% efficiency) completely blocked the Pg-induced 1.6-fold increase in nuclear p27 (**Figure 6B** **, right panels**) and the 30% growth inhibitory effect (**Figure 6C**). Moreover, proliferation was partially blocked in the untreated and Pg-treated Cdh1 siRNA transfected cells. Whereas Pg caused a decrease in nuclear Skp2 and Cks1 (**Figures 5B** **,** **6B**), the lack of Cdh1 E3 ligase activity in the knock-down cells, expectedly increases the basal levels of nuclear Skp2 and Cks1 (**Figure 6B** **, right panels**). In addition, Pg-treatment decreased cytoplasmic Skp2 in both the control siRNA and Cdh1 siRNA by 51% and 45%, respectively (**Figure 6B** **, left panels**). These data provide strong support for a mechanism by which PR-mediated Pg action increases nuclear p27 for inhibition of proliferation by raising Cdh1 in the nucleus, which in turn degrades Cks1 and Skp2, as evidenced by their increase in the presence of lactacystin and Pg [plus E2](**Figure 5B**).

### Estrogen and progesterone have opposite effects on p27 and proteins of the ubiquitin-proteasome system in primary endometrial epithelial cells

**Figure 7:**
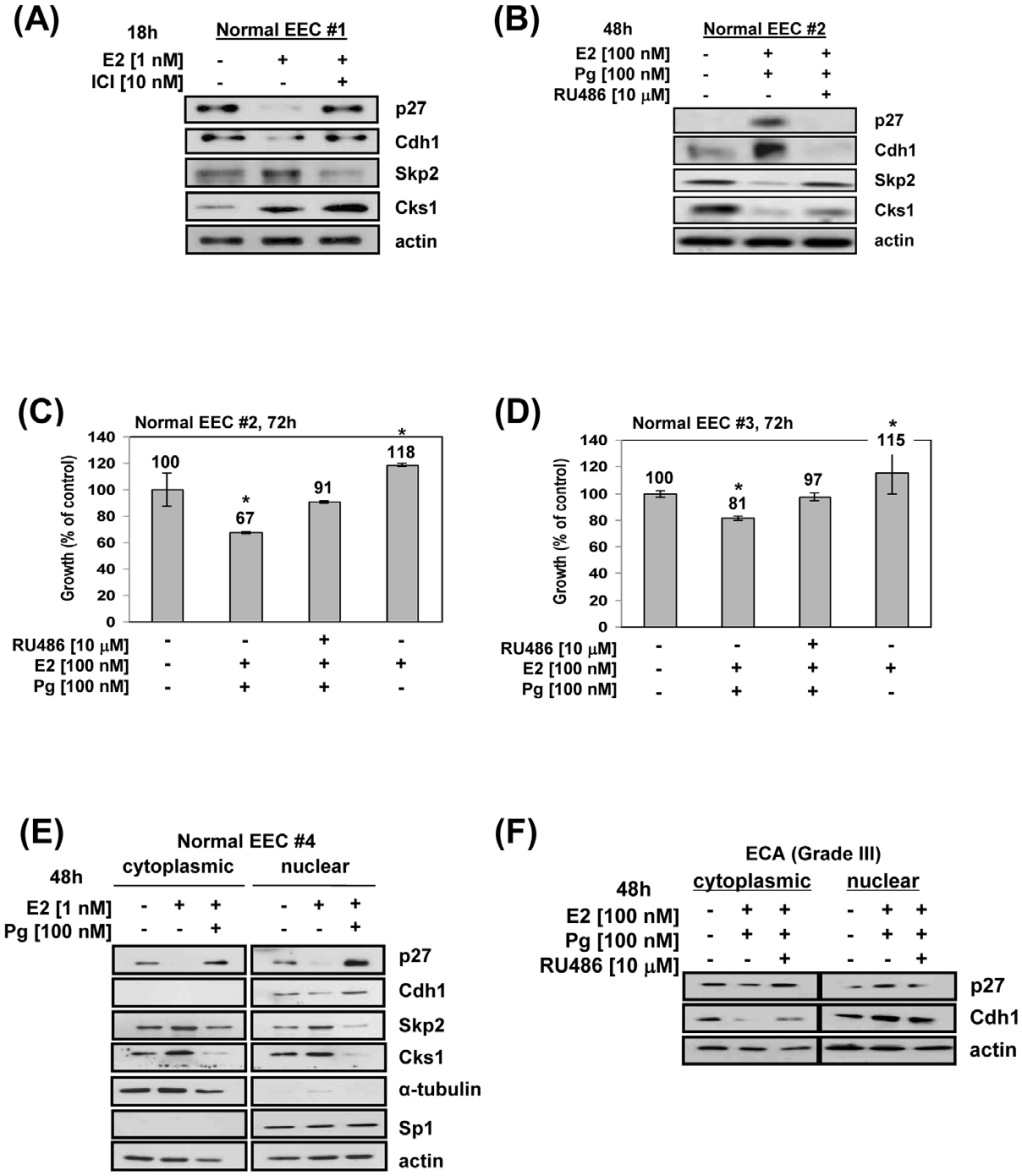
Identical responses to E2 and Pg by ECA cell lines and endometrial epithelial cells (EECs). A, E2; B, Pg treatment of primary EECs: normal EECs from proliferative phase were treated with either E2 without or with ICI or treated with Pg/E2 with or without RU486, cell lysates prepared and immunoblotted for protein levels shown as described in Materials and Methods. A and B are different patients showing different basal levels of p27 and Cdh1. C, D: E2, E2 plus Pg treatment, cell proliferation (by MTS assay): parallel experiment for patient #2, as described in Materials and Methods; *p<0.05. E, E2, Pg treatment, subcellular fractionation: Primary EECs from proliferative phase were treated with E2 or Pg/E2, subjected to subcellular fractionation, and immunoblot analysis for the protein levels shown and performed as described in Materials and Methods. F, Pg, Primary ECA cells (grade III): Cells were isolated, treated with Pg/E2 in the presence or absence of RU486, subjected to subcellular fractionation followed by immunoblot analysis for Cdh1 and p27, as described in Materials and Methods.

EECs from endometrial tissue yielded identical responses for p27, Cdh1, Skp2 and Cks1 as shown for the ECC-1 cell line following treatment with E2 and Pg. Specifically, E2 via the ER decreased p27 by 88% (**Figure 6A**) and Cdh1 by 71% and increased Skp2 and Cks1 by 1.2 and 2.3-fold, respectively. In contrast, Pg-treatment of primary EECs increased p27 and Cdh1 by 2.7-fold and 1.8-fold, respectively, whereas Skp2 and Cks1 were both decreased by 69% (**Figure 7B**). Treatment of primary cells with E2 stimulated growth by 18% and 15% whereas Pg (plus E2) inhibited growth by 33% and 29%, which was nearly fully blocked by RU486 (**Figure 7C and 7D**) in both patients. Nuclear-cytoplasmic fractionation of EECs from one normal endometrial tissue revealed that Cdh1 was only present in the nucleus and that E2 decreased Cdh1 by 41%, decreased p27 mainly in the nucleus (by 50%), increased Skp2 in both fractions but mostly in the nucleus (1.3-fold), and increased Cks1 in both fractions but with more in the cytoplasm (1.5-fold; **Figure 7E**). In direct contrast, Pg predominantly increased nuclear p27 by 2.6-fold and nuclear Cdh1 by 1.3-fold whereas Skp2 was decreased in the nucleus and cytoplasm by 41%, and 89%, respectively. Pg reduced Cks1 by 50% in the nucleus and less in the cytoplasm. Interestingly, whereas Cdh1 was not found in the cytoplasm in the ECC-1 cell line and primary normal EECs (n = 3), Cdh1 was detected in the cytoplasm in primary ECA cells (n = 3, grades II, III; **Figure 7F**), which was decreased by 44% in this subcellular fraction while it was increased in the nucleus by 44%, following Pg treatment. The same pattern was shown for p27, which was present in the cytoplasm and increased in the nucleus by 1.4-fold in response to Pg. The consistent responses to E2 and Pg observed among the four normal primary EECs not only implicate that consistency can be obtained despite patient heterogeneity but suggest that the hormone responses we observed in the immortalized cell lines are likely physiological.

## Discussion

**Figure 8:**
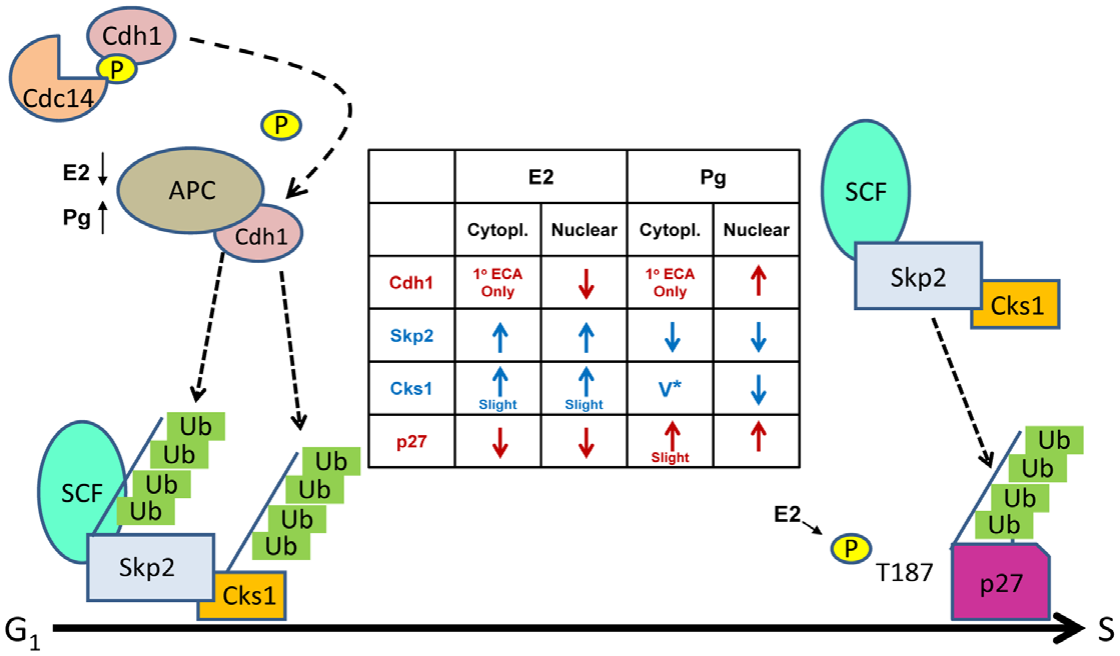
Estrogen (E2) and progesterone (Pg) inversely regulate the levels of nuclear p27 and Cdh1, Skp2, and Cks1 proteins of the ubiquitin-proteasome system for cell cycle control. Cdc14 phosphatase keeps Cdh1 bound to APC; this is the E3 ligase that ubiquitylates Skp2/Cks1 of the SCF complex. E2 decreases nuclear [APC]Cdh1 and induces phosphorylation of p27 on T187 signaling its ubiquitylation by [SCF]-Skp2/Cks1. The decrease in [APC]Cdh1 prevents ubiquitin-mediated degradation of Skp2/Cks1 allowing [SCF]Skp2/Cks1 to ubiquitylate p27 causing its proteasomal degradation; cells progress through S phase. Pg increases [APC]Cdh1, which ubiquitylates [SCF]Skp2/Cks1 causing their degradation blocking p27 degradation; cells are blocked in G1. Ovals are E3 ligases; green small squares are chains of ubiquitin (Ub); small yellow circles indicate phosphorylation (P). Center Table: Slight (<15%); v* = variable, slightly up in ECC-1 and variable EECs. Cdh1 was not present in the cytoplasm in ECC-1 cells or EECs but was found in primary ECA cells (n = 2/3).

The present studies provide new insights into hormone regulation of cell proliferation. Specifically, we describe novel mechanisms by which E2 and Pg have opposite effects on endometrial growth through the ubiquitin-proteasome system to regulate the levels of nuclear p27, important for G1 arrest (**Figure 8**). We conclude that: 1) the levels of the Cdh1 component of the APC/Cdh1 and p27 vary directly and are inverse to the Skp2 and Cks1 components of the SCF-Skp2/Cks1 in response to E2 and Pg with stimulatory and inhibitory effects, respectively; 2) the regulation of Cdh1, Skp2, and Cks1 of the UPS and p27 is not at the level of transcription; 3) the ECA cell lines used herein are appropriate paradigms for this study since their responses to E2 and Pg were identical to primary EECs. Specifically, we show that E2 has two major effects: 1) induces MAPK/ERK2-dependent phosphorylation of p27 on T187, which only occurs in the nucleus and thus, targets the ubiquitylation and degradation of p27 by SCF-Skp2/Cks1 shown to take place in this subcellular compartment [7], [9], [30] 2) causes a decrease in Cdh1 to prevent degradation of Skp2 and Cks1; this raises the levels of Skp2/Cks1 causing degradation of p-p27(T187) for stimulation of cell proliferation. The decrease in p27 stability induced by E2 is underscored by a 1.5 h faster turn-over rate at a 6 h time point following treatment with CHX, which was commensurate with an increase in Skp2 by 1.3 fold. Conversely, Pg has the full opposite effect as it increases Cdh1 and its binding to the APC/Cdh1 for degradation of Skp2 and Cks1 thus, leaving nuclear p27 intact for G1 arrest. To substantiate this effect, Pg increased the stability of p27 protein as the levels were increased by 1.5-fold at 6 h after treatment with CHX whereas Skp2 turn-over was decreased by 6.2 h. The decrease in Skp2 was at least, in part, due to the degradation of Skp2 by the Pg-induced increase in Cdh1. The effects of E2 and Pg on the levels of p27, Skp2, Cks1, and Cdh1 are dose and time-dependent and are consistent with their expected effect on cell cycle distribution. Importantly, knocking-down Skp2 completely obviates both E2-induced degradation of nuclear p27 and stimulation of cell proliferation. Therefore, E2-induced Skp2 E3 ligase activity is required for nuclear p27 degradation and involves a functional interaction between Skp2/Cks1 and p27. These data provide proof of principle for the role of E2 in the pathogenesis of ECA and explain the action of progestins (e.g., Megace® etc) as successful therapeutic agents for AEH and ECA in vivo [4], [5]. Studies show that an increase in nuclear p27 predicts positive outcomes from Pg therapy [20], [34] underscoring p27 in the physiological regulation of endometrial growth and as a significant molecular target for ECA. As perpetual proteasomal degradation of p27 is an early event in ECA oncogenesis [6], the current studies implicate that blocking proteasomal degradation of nuclear p27 to regain growth control is a rational preventative and therapeutic approach to ECA particularly, in patients lacking PR. However, p27 can be mislocalized to the cytoplasm in ECA [18], [19] and other human cancers thereby abolishing its function as a Cdk inhibitor [7], [15], [17], [35]. Phosphorylation of p27 on Ser 10 by kinase interacting stathmin (KIS) causes its binding to CRM-1 for exportation to the cytoplasm [7], [12], [17]. In addition, a high frequency of PTEN inactivating mutations in type I ECA (over 50%) leads to increased Akt activity [1]; Akt phosphorylates p27 on Thr157 [13], [36] blocking its nuclear import. Interestingly, PTEN and p27 are both decreased with a concomitant increase in Skp2 [37], [38], [39]. In the cytoplasm, p27 can be further phosphorylated on, T198 by AGC kinase, downstream from PI3K-Akt stabilizing the molecule in this cellular compartment [14]. Cytoplasmic p27 represses RhoA thereby affecting cytoskeleton dynamics with effects on migration and metastasis [7], [16], [17]. As we show an increase in p27 mainly in the nucleus in response to Pg, Pg therapy might increase p27 shuttling into the nucleus.

E2 and Pg treatment induced the expected growth stimulatory and inhibitory effects respectively, in vitro on both human ECC-1 cells and primary EECs. The response levels to these hormones with respect to growth are consistent with those obtained by others in vitro in the absence of peptide growth factors [40], [41], [42]. In addition, it is well-known that in vivo, hormone-induced growth responses rely in part on the hormone receptor-driven release of polypeptide growth factors and chemokines from stromal cells [43], [44]. To this point, in vivo, using human EECs and mouse or human stromal cells as recombinant tissues, we have shown that E2 induces a strong mitogenic response in the epithelial cells within tissue explants [43]. A recent study shows that endometrial stromal cells release FGF to stimulate proliferation of EECs in response to E2 [41]. In addition, soluble mediators including the growth inhibitor TGF-β, released from the stroma in response to Pg, would contribute to the growth inhibitory effect of Pg, in vivo [29]. Interestingly, in experiments showing that p27 is required for growth inhibition by TGF-β, we obtained 55% and 65% growth inhibitory response in EECs and ECC-1 cells, respectively, with a similar 2-fold increase in the levels of nuclear p27 in HEC-1A [6], [45] as we show in the current study. Nonetheless, aberrant cell cycle progression and continuous cycling is a hallmark of tumorigenesis and proteasomal destruction of p27 by SCF-Skp2/Cks1 appears to be a common mechanistic failure in ECA and other cancers [7], [17], [20], [46].

The E3 ligase activity of Cdh1 bound to APC is in a complex of at least 11 core subunits and has different functions during the cell cycle [33], [47]. In late mitosis, Cdh1/APC prevents premature exit from mitosis before sister chromatid separation. The APC/Cdh1 also promotes exit from mitosis by causing degradation of mitotic cyclins and maintains G1 by its E3 ligase activity for Skp2 and Cks1, which increases nuclear p27 [26], [27]. The action and binding partners of APC are controlled by multiple phosphorylation, phosphatase and ubiquitylation events on specific sites of which the functional effects are largely unknown [31], [32], [33]. Nonetheless, the phosphatase Cdc14 maintains Cdh1 in a hypophosphorylated state that keeps APC tightly bound to Cdh1 for its activation but, Cdh1 is dissociated following its phosphorylation by Cdk1/2 and becomes a substrate for an SCF complex while APC binds Cdc20 (APC/Cdc20) for mitotic transitions [27], [32], [33]. Stabilization of Skp2 is achieved by phosphorylation on Ser64, which prevents its binding to the APC/Cdh1 for destruction [30], [48]. We show here that E2 decreases and Pg increases Cdh1 varying directly with the levels of p27 as its apparent ultimate master regulator. Furthermore, Cdh1 is the critical nexus for the marked increase in p27 by Pg as shown by depletion of Cdh1 by siRNA, which completely obviates the Pg-induced increase in nuclear p27 and growth inhibitory response. This relationship is recapitulated in studies showing that absence of Cdh1 caused an increase in Skp2 and increased breast tumor growth in a xenograft model whereas elevation of Cdh1 had the opposite effect [49]. It is notable that lactacystin blocks E2-induced degradation of Cdh1 suggesting that an upstream, still to be identified, E3 ligase for APC/Cdh1 such as an SCF E3 ligase [27] or APC/Cdh1 itself [31], [50], is involved in the regulation/decrease of Cdh1 by E2 via proteasomal degradation. Our studies open a new question concerning identifying the upstream effectors of hormonal regulation of Cdh1 to control the levels of p27.

Whereas the effect of E2 or Pg on Cdh1 levels occurs only in the nucleus in ECC-1 cells and EECs, E2 increases and Pg decreases Skp2 levels in both the cytoplasm and nucleus and furthermore, knocking down Skp2 preserves p27 levels and blocks E2-induced degradation of p27 in both the cytoplasm and nucleus. The presence of Skp2 in the cytoplasm, due to its phosphorylation on Ser72 by Akt in its putative NLS, has been controversial [30], [51], [52], [53]. The E3 ligase KPC, not Skp2, is ostensibly responsible for causing degradation of p27 in the cytoplasm. Our data suggest that Skp2 might indeed exert E3 ligase activity on p27 in both cellular compartments. Interestingly, Cdh1 was found in the cytoplasm only in primary ECA cells (n = 2/3). Furthermore, Pg treatment of these cells increased Cdh1 and p27 in the nucleus while decreasing these proteins in the cytoplasm. This suggests that the therapeutic action of Pg might not only be by increasing the levels of nuclear p27 but also by inducing shuttling of Cdh1 and p27 from the cytoplasm to the nucleus where Cdh1 can protect p27 from degradation by SCF-Skp2/Cks1 to block Cdk2.

Our studies show that E2 and Pg act in an opposing manner as physiological molecular switches that exert their growth regulatory effects on the endometrium by utilizing ubiquitin-mediated protein degradation of p27 and proteins of the UPS. Therefore, normal and malignant endometrial cells provide a useful platform for understanding how these hormones regulate the levels of cell cycle proteins via the UPS. Steroid regulation of gene expression occurs by both genomic and non-genomic signaling mechanisms [54]. Genomic signaling, which can be rapid or slow, involves a ligand-activated direct interaction of the DNA-binding domain (DBD) in the hormone receptor on specific hormone response elements (RE) on target genes (i.e., ER binds to ERE and PR to PRE). For example, the ER binds directly to, and activates the cyclin D1 gene [55]. Non-genomic signaling is generally rapid without congruent transcription, which occurs later for functional effects. Signaling can be initiated by cell surface membrane receptors including hormone receptors and may involve interactions of the nuclear steroid receptors with membrane and cytoplasmic signaling pathways such as G proteins and protein kinases that use for example, SP-1 or AP-1 sites on target genes [56], [57], [58]. Both means of hormone signaling can affect an array of genes involved in cell proliferation. Furthermore, post-translational modification by phosphorylation or sumoylation of PR and ERα dictates differential gene activation [56], [59] and a recent study shows that ERα is subject to E2-dependent proteolysis through an SCF-Skp2 binding mechanism [60]. The effects of E2 and Pg on the Cdh1-Skp2/Cks1 axis that regulate p27 protein levels shown here, which are abrogated in the presence of ER and PR antagonists are categorically non-genomic. However, the effects of hormone signaling appear to be unconventional (i.e., not rapid) as neither E2 nor Pg affected Cdh1, Skp2, Cks1 or p27 transcription in ECC-1 cells within 12–24 h. Rather, our data suggest that estrogen and progesterone regulation of growth in part occurs by post-translational protein modifications involving phosphorylation and ubiquitylation events. Importantly, our analysis of the Cdh1-Skp2/Cks1 axis that regulates the levels of p27, as our target focus must occur concurrently in the presence of classic genomic and non-genomic effects related to hormone signaling by ER and PR for growth regulation and other E2 and Pg-induced cellular responses.

In conclusion, preventing degradation of nuclear p27 for normal growth control can be approached by blocking p27 degradation by Skp2/Cks1 as a potential druggable target [22]. However, SCF has different F-box substrate recognition modules for both tumor suppressors, such as p27, p57, and p21 and oncogenes, such as Cyclin D1, mTOR, and c-Myc [25]. Skp2 is the F-box substrate recognition module that forms a structurally active site in the presence of Cks1 with ubiquitylation specificity for only p27 and p21 [22], [23]. Therefore, a specific E3 ligase inhibitor of Skp2/Cks1might be an optimal approach to specifically raise nuclear p27 with an obligate need to inhibit only nuclear p27 degradation. A second approach is to increase Cdh1 as this protein appears to be localized in the nucleus in ECC-1 and EEC cells. Further studies are necessary to determine the mechanisms involved in the regulation of Cdh1 by Pg for therapeutic purposes and by E2, to further an understanding of endometrial carcinogenesis.

## Materials and Methods

### Cell culture and treatments

Fresh human endometrial tissue was obtained from hysterectomies according to NYU institutional review board (IRB) protocol including obtaining written consent from all patients: written consent was approved by NYU IRB- Board A (IRB# H9243; Approved Oct 14, 2009–Aug 28, 2012). The endometrial carcinoma cell lines ECC-1 (well differentiated), HEC-1B (moderately differentiated), and KLE (poorly differentiated) were from American Tissue Culture Collection. Cells were seeded at 3×10^5^/well/6-well plate and incubated in Minimum Essential Medium (Invitrogen) supplemented with 10% FBS (BioWest), 1 mM sodium pyruvate (Invitrogen) and 2 mM L-Glutamine (Invitrogen) for HEC-1B cells and DMEM/F-12 (1∶1) (Invitrogen) with 10% FBS for ECC-1 and KLE cells. Primary endometrial epithelial (EECs; from leiomyomas) or carcinoma (ECA) cells were isolated from fresh hysterectomy tissue procured from New York University-Tisch and Bellevue Hospitals according to NYU Institutional Review Board protocol (IRB# H9243), as described [61]. EECs were plated at 4–6×10^6^ cells per 6-cm Primaria® dish (BD Falcon) and cultured in McCoy's 5A (US Biological) complete medium containing 10% charcoal-stripped FBS. For all treatments except experiments using cycloheximide (CHX) treatment, cells were cultured to 70–80% confluence, synchronized in serum-free media for 24 h and treated with 17β-estradiol (E2, 0.1–100 nM) or medroxyprogesterone 17-acetate (Pg; 1–100 nM, Sigma) in the presence of 100 nM E2 (i.e., to mimic the menstrual cycle and thereby increase progesterone receptors [PR] for a robust Pg-induced response, as previously shown [6], [28], [29] for the times shown. For p27 and Skp2 turn-over/stability studies using CHX, ECC-1 cells were grown to 50% confluence, synchronized as above, treated with E2 (1 nM) or Pg (100 nM) plus E2 (100 nM) for 18 h and 48 h, respectively or left untreated for the same time periods. CHX (20 µM) was added at 6 h prior to final cell harvest. Cell lysates were prepared at 0, 1, 2, and 6 h for immunoblotting. Certain experiments included estrogen receptor (ER) antagonist, 10 nM ICI 182,780 (ICI; Zeneca Limited) or a PR antagonist, 10 µM Mifepristone (RU-486; Sigma Chem), the proteasome inhibitor, 1 µM Lactacystin (Lac; Calbiochem), and the MAPK/MEK inhibitors, 20 µM PD98059 and 20 µM U0126 (both from Calbiochem).

### Cell proliferation Assay and cell cycle distribution

Primary EECs or ECC-1 cells were seeded onto 96-well plates (BD Biosciences) at a density of 3×10^4^ and 4×10^3^ cells/well, respectively, cultured until 50% or 70% confluent for E2 or Pg treatments, respectively, cells synchronized in serum-free media for 24 h, and treated in triplicate with E2 (0.1–100 nM) or Pg (1–100 nM, in the presence of 100 nM E2) for the times indicated. Cell proliferation was determined by the MTS assay, as described [6], [45]; values are expressed as per cent of untreated controls (100%) and presented as mean±SD. The student *t* test was used to predict statistical significance among treatments to generate two-tailed p values with significance as *P≤0.05. Cell cycle distribution was performed using propidium iodide staining, analyzed by FACS, and presented as percent of cells in phases of the cell cycle [61].

### Western blotting

Following each experiment, either whole cell extracts of primary EECs and the cell lines ECC-1, HEC-1B, and KLE or nuclear cytoplasmic fractionations of ECC-1 cells were obtained, as described [45]. Equal protein concentrations (BCA assay; ThermoScientific) were applied (20 µg for whole cell extracts, 10 or 5 µg for cytoplasmic/nuclear extracts) to either 12.5% or 5–20% polyacrylamide SDS-PAGE and immunoblotting performed exactly as described [45]. Primary antibodies included: mouse anti-human p27^Kip1^ (1∶1000, Clone 57, BD Transduction Laboratories), rabbit anti phospho-p27 (1∶1000, PT187, Invitrogen), mouse anti-human p45/Skp2 (1∶1000, 8D9, Invitrogen), rabbit anti-human Cks1 (1∶500, C-term, Invitrogen), and mouse anti-human Cdh1 (1∶1000, DH01, Calbiochem). Mouse anti-human-pERK1/2 (1∶1000, E4), rabbit anti-human ERK1/2 (1∶1000, K-24), mouse anti-human Cdc27 (1∶500, AF3.1) were from Santa Cruz Biotechnology. For p27, Skp2, Cks1, Cdh1 and Cdc27, the membranes were blocked with 5% non-fat dry milk in TBS containing 0.1% Tween-20 (TBST) for 1 h. For phospho-p27 antibody, the membrane was blocked in 3% BSA in TBST. The membranes were incubated with primary antibodies prepared in TBST at 4°C overnight. The blots were stripped and reprobed with mouse anti-β-actin (1∶10000, AC-15, Sigma) for total cell lysates, and for purity of cytoplasmic and nuclear fractions, with mouse anti-α-tubulin (1∶10000, B-5-1-2, Sigma) and rabbit anti-Sp1 (1∶1000, H-225, Santa Cruz), respectively. Secondary antibody was peroxidase-conjugated goat anti-mouse or anti-rabbit IgG (1∶2000, ThermoScientific) prepared in TBST. Protein bands were resolved with the SuperSignal West Dura kit (ThermoScientific), densitometry was performed on every blot, each protein band normalized to β-actin and compared with untreated control. Fold increase or per cent decrease for each protein response derived from the densitometric scanning is presented in the text. Exposure times on x-ray films were adjusted for optimal visualization and quantification of protein bands by densitometry. Blots are representative of a minimum of three independent experiments (n = 3).

### RNA interference

ECC-1 cells were seeded at 1.5×10^5^ cells/well for ERK1, ERK2 and 1.2×10^5^ cells/well for Skp2 and Cdh1 knock-down in 12-well plates in DMEM/F-12 supplemented with 10% FBS and 24 h later, cells were transfected with either control siRNA (Santa Cruz) or 25 nM ERK1, ERK2 siRNA (Dharmacon), or 10 nM of *fzr* (Cdh1) siRNA or *Skp2* siRNA (both Santa Cruz), using HiPerfect transfection reagent (Qiagen); each with 2 different specific siRNAs (not shown) and then finally using a pool of 3 siRNAs (as presented). After 24 h, the cells were synchronized and treated with hormones and inhibitors, as described above, and total cell lysates quantified for knock-down efficiency by immunoblotting and densitometry. For subcellular fractionation, ECC-1 cells were seeded at 3.5×10^5^ and treated and analyzed as above. For companion cell proliferation experiments, the Skp2 knock-down and Cdh1 knock-down cells were seeded at 1×10^4^/well and 4×10^3^/well, respectively in 96-well plates, the cells transfected, as above, synchronized in serum-free media, treated with hormones and cell proliferation determined, as above.

### Cdh1 binding to APC by immunoprecipitation

ECC-1 cells, in complete media and seeded in 10 cm tissue culture dishes, were synchronized and treated with 100 nM of each E2 and Pg for 48 h. 750 µg of protein in 0.5 ml RIPA buffer was first precleared with 0.75 µg mouse IgG for 30 min followed by 20 µl of Protein A/G PLUS-Agarose beads (Santa Cruz) for 1 hr. Precleared lysates were incubated with 0.75 µg mouse anti-Cdh1 or 0.75 µg mouse IgG overnight and then with 20 µl of Protein A/G PLUS-Agarose beads for 1 h at 4°C with end-over rotation. The beads-immunoprecipitate complex was washed in cold RIPA buffer, re-suspended in Laemmli buffer, and equal volumes subjected to 12.5% SDS-PAGE, and immunoblotted with anti-Cdc27. Control: 10 µg of total lysate (input).

### Quantitative RT-PCR

Cells were seeded, treated and harvested at indicated time points, total RNA isolated using the RNeasy Mini Kit (Qiagen), and first strand cDNA synthesized with 0.5 or 1 µg total RNA using the QuantiTect Reverse Transcription kit (Qiagen). Quantitative PCR was performed using the iQ SYBR Green Supermix (Bio-Rad) with the primers shown (Table S1, Supplementary Data) in a Mx3000P QPCR System (Stratagene). PCR conditions for all genes were as follows: 95°C for 3 min, followed by 40 cycles of 95°C for 30 sec, 55°C for 30 sec and 72°C for 30 sec. Melting curve analysis was performed to ensure primer specificity. Relative gene expression was calculated against actin as control using the ΔΔC^T^ method.

## Supporting Information

Figure S1 **Estrogen (E2)-induced decrease of p27 is mediated by ERK-dependent phosphorylation on Thr187.** (A) The endometrial carcinoma cell line, HEC-1B (moderately differentiated; from ATCC) in Minimum Essential Medium (Invitrogen) supplemented with 1 mM sodium pyruvate and 2 mM L-Glutamine and 10% FBS were seeded at 3×10^5^ cells/well/6-well plate and cultured until 70% confluency. The cells were synchronized in serum-free media for 24 h and treated with 0–100 nM 17β-Estradiol (E2) for 18 h. Cell lysates were prepared in cold RIPA buffer (50 mM Tris-HCl, 150 mM NaCl, 1 mM NaF, 1 mM Na_3_VO_4_, 0.25% sodium deoxycholate, 1% NP40 and 1 mM EDTA, pH 7.4) supplemented with 1 mM PMSF and protease inhibitor cocktail (Sigma). Protein concentrations were determined by MicroBCA Protein Assay kit (ThermoScientific) and equal protein concentrations (20 µg/well) were applied to an SDS-PAGE (12% acrylamide) and then transferred to nitrocellulose membranes. The membranes were blocked with 3% BSA in TBS containing 0.1% Tween (TBST) for 1 h and incubated with rabbit anti-phospho-p27 (1∶1000, PT187, Invitrogen) in TBST overnight at 4°C followed by peroxidase conjugated goat anti-rabbit secondary antibody (1∶2000) in TBST for 1 h. The blots were incubated with SuperSignal West Dura Extended Duration Substrate kit (ThermoScientific) and protein bands visualized by exposure on x-ray film (Denville Scientific). The blots were stripped and re-probed with anti-β-actin (1∶10,000, AC-15, Sigma). Densitometry was performed and the intensity of each band determined by ID Image analysis (Kodak); each protein band was normalized to the density of actin in each well and expressed as fold-increase or per cent decrease compared to the untreated control. The blot shows that E2 dose-dependently increases the phosphorylation of p27 at T187 (p-p27[T187]) with a peak response of 1.75 fold at 0.1 nM E2 compared to the untreated control that decreases by 40% at 10 nM. The HEC-1B cell line was more sensitive to E2 than the ECC-1 cell line. (n = 2 for HEC-1B cells). (B) Estrogen (E2)-induced decrease in p27 is mediated by MAPK/MEK. The endometrial carcinoma cell line, ECC-1 cells, were seeded, synchronized and treated as described for Supplemental Figure 1A, in the presence or absence of the MAPK/MEK inhibitor, PD98059 (PD), (Calbiochem) at a final concentration of 20 µM, for 18 h. Cell lysates were prepared and equal protein concentrations (20 µg) subjected to SDS-PAGE followed by protein transfer to nitrocellulose membranes, as described in Supplemental Figure 1A. The membranes were blocked with 5% non-fat dry milk in TBST for 1 h followed by incubation overnight with mouse anti-human p27^kip1^ (1∶1000, Clone 57, BD Transduction Labs) in TBST followed by peroxidase-conjugated goat anti-mouse secondary antibody (1∶2000, ThermoScientific). The intensity of p27 protein band was determined as described in Supplemental Figure 1A. The immunoblot shows that E2 decreases p27 in total cell lysates by 46% of the untreated control, which is partially blocked by the MAPK/MEK inhibitor PD 98059 (24% less than control). (n = 3 separate experiments).
(PDF)

Figure S2 **Knocking-down Skp2 (Skp2 siRNA) blocks the estrogen (E2)-induced decrease in p27 and markedly increases the level of p27.** ECC-1 cells were seeded at 1.2×10^5^ in 12-well plates in DMEM/F12 supplemented with 10% FBS. 24 h later, the cells were transfected with either control siRNA (Santa Cruz Biotechnology) or *Skp2* siRNA (pool of 3 siRNAs) using HiPerfect transfection reagent (Qiagen). After 24 h, the cells were synchronized and treated for 18 h with 1 nM E2 or 1 nM E2 plus 1 µM Lactacystin (Lac; proteasome inhibitor) or 1 nM E2 plus 10 nM ICI 182,780 (ICI; E2 antagonist), total cell lysates prepared in cold RIPA buffer, and 20 µg of total protein analyzed for Skp2 and p27 levels. The membranes were blocked with 5% non-fat dry milk in TBST for 1 h and incubated overnight at 4°C with mouse anti-human p45/Skp2 (1∶1000, 8D9, Invitrogen) or mouse anti-human p27 (BD Transduction Labs; membranes were cut a the appropriate anticipated molecular weight and incubated separately), as described for each antibody in Materials and Methods, followed by peroxidase conjugated goat anti-mouse IgG (1∶2000) in TBST for 1 h. The blots were reprobed with anti-actin and bands quantified as described in Supplemental Figure 1A. The blot shows that in the control siRNA transfected cell, E2 decreases p27 by 50%, which is completely blocked by Lac and nearly completely blocked by ICI compared to the untreated control. In the Skp2 siRNA transfected cells, the p27 levels were greatly increased as shown by the 2 min compared to 5 sec exposure. Importantly, p27 levels remained unchanged following treating the cells with E2 (n = 3 separate experiments).
(PDF)

Table S1 **List of primers used for real-time RT-PCR.**
(PDF)
