# Supplementary figures and images for: Estrogen and Progesterone Regulate p27kip1 Levels via the Ubiquitin-Proteasome System: Pathogenic and Therapeutic Implications for Endometrial Cancer
Source: PLoS One. 2012 Sep 27;7(9):e46072. doi: 10.1371/journal.pone.0046072 (PMC3459846; doi:10.1371/journal.pone.0046072)

Figure S1

(A) HEC-1B

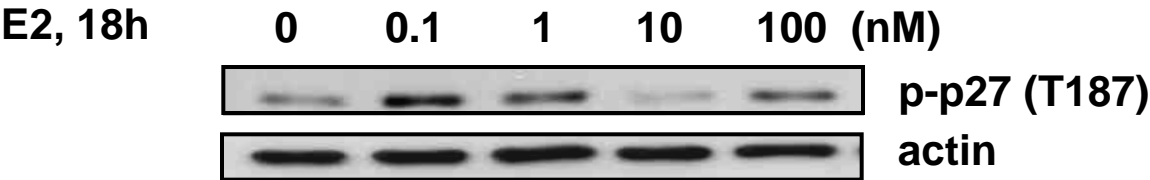

(B) ECC-1

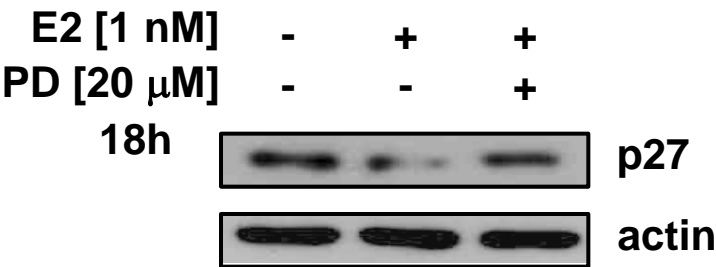

Supplement: Figure S1 — Estrogen (E2)-induced decrease of p27 is mediated by ERK-dependent phosphorylation on Thr187. (A) The endometrial carcinoma cell line, HEC-1B (moderately differentiated; from ATCC) in Minimum Essential Medium (Invitrogen) supplemented with 1 mM sodium pyruvate and 2 mM L-Glutamine and 10% FBS were seeded at 3×10^5^ cells/well/6-well plate and cultured until 70% confluency. The cells were synchronized in serum-free media for 24 h and treated with 0–100 nM 17β-Estradiol (E2) for 18 h. Cell lysates were prepared in cold RIPA buffer (50 mM Tris-HCl, 150 mM NaCl, 1 mM NaF, 1 mM Na3VO4, 0.25% sodium deoxycholate, 1% NP40 and 1 mM EDTA, pH 7.4) supplemented with 1 mM PMSF and protease inhibitor cocktail (Sigma). Protein concentrations were determined by MicroBCA Protein Assay kit (ThermoScientific) and equal protein concentrations (20 µg/well) were applied to an SDS-PAGE (12% acrylamide) and then transferred to nitrocellulose membranes. The membranes were blocked with 3% BSA in TBS containing 0.1% Tween (TBST) for 1 h and incubated with rabbit anti-phospho-p27 (1∶1000, PT187, Invitrogen) in TBST overnight at 4°C followed by peroxidase conjugated goat anti-rabbit secondary antibody (1∶2000) in TBST for 1 h. The blots were incubated with SuperSignal West Dura Extended Duration Substrate kit (ThermoScientific) and protein bands visualized by exposure on x-ray film (Denville Scientific). The blots were stripped and re-probed with anti-β-actin (1∶10,000, AC-15, Sigma). Densitometry was performed and the intensity of each band determined by ID Image analysis (Kodak); each protein band was normalized to the density of actin in each well and expressed as fold-increase or per cent decrease compared to the untreated control. The blot shows that E2 dose-dependently increases the phosphorylation of p27 at T187 (p-p27[T187]) with a peak response of 1.75 fold at 0.1 nM E2 compared to the untreated control that decreases by 40% at 10 nM. The HEC-1B cell line was more sensitive to E2 than t [file pone.0046072.s001.pdf]

Figure S2

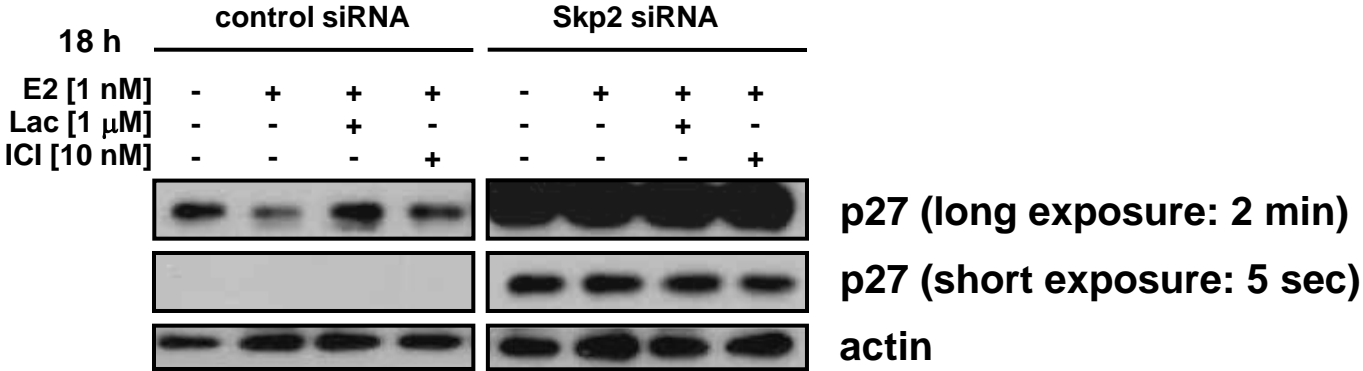

Supplement: Figure S2 — Knocking-down Skp2 (Skp2 siRNA) blocks the estrogen (E2)-induced decrease in p27 and markedly increases the level of p27. ECC-1 cells were seeded at 1.2×10^5^ in 12-well plates in DMEM/F12 supplemented with 10% FBS. 24 h later, the cells were transfected with either control siRNA (Santa Cruz Biotechnology) or Skp2 siRNA (pool of 3 siRNAs) using HiPerfect transfection reagent (Qiagen). After 24 h, the cells were synchronized and treated for 18 h with 1 nM E2 or 1 nM E2 plus 1 µM Lactacystin (Lac; proteasome inhibitor) or 1 nM E2 plus 10 nM ICI 182,780 (ICI; E2 antagonist), total cell lysates prepared in cold RIPA buffer, and 20 µg of total protein analyzed for Skp2 and p27 levels. The membranes were blocked with 5% non-fat dry milk in TBST for 1 h and incubated overnight at 4°C with mouse anti-human p45/Skp2 (1∶1000, 8D9, Invitrogen) or mouse anti-human p27 (BD Transduction Labs; membranes were cut a the appropriate anticipated molecular weight and incubated separately), as described for each antibody in Materials and Methods, followed by peroxidase conjugated goat anti-mouse IgG (1∶2000) in TBST for 1 h. The blots were reprobed with anti-actin and bands quantified as described in Supplemental Figure 1A. The blot shows that in the control siRNA transfected cell, E2 decreases p27 by 50%, which is completely blocked by Lac and nearly completely blocked by ICI compared to the untreated control. In the Skp2 siRNA transfected cells, the p27 levels were greatly increased as shown by the 2 min compared to 5 sec exposure. Importantly, p27 levels remained unchanged following treating the cells with E2 (n = 3 separate experiments). (PDF) [file pone.0046072.s002.pdf]
