# Supplementary material for: Estrogen and Progesterone Regulate p27kip1 Levels via the Ubiquitin-Proteasome System: Pathogenic and Therapeutic Implications for Endometrial Cancer
Source: PLoS One. 2012 Sep 27;7(9):e46072. doi: 10.1371/journal.pone.0046072 (PMC3459846; doi:10.1371/journal.pone.0046072)
Supplement: Table S1 — List of primers used for real-time RT-PCR. (PDF) [file pone.0046072.s003.pdf]

Table S1

Primers used for real-time RT-PCR

Primer Name	Accession Number	Sequence
p27 F	NM_004064	5’-CTTGCCCGAGTTCTACTACAGAC-3’
p27 R		5’-CAAATGCGTGTCCTCAGAGTTAG-3’
Skp2 F	NM_005983	5’-TCAACTACCTCCAACACCTATCAC-3’
Skp2 R		5’-GGTACCATCTGGCACGATTCC-3’
Cks1 F	NM_001826	5’-GAGTATCGACATGTCATGCTGC-3’
Cks1 R		5’-TCTTTGGTTTCTTGGGTAGT-3’
Cdh1 F	NM_016263	5’-GTCCAAGCACGCCAACGAGCTGGTGA-3’
Cdh1 R		5’-GACACAGACTCCTTTGTCGAACGG-3’
PR F	NM_000926	5’-GGTCTACCCGCCCTATCTCA-3’
PR R		5’-GGCTTGGCTTTCATTTGGAA-3’
glycodelin F	NM_002571	5’-CACGCTGCTCGATACTGACTACGAC-3’
glycodelin R		5’-TGGAGGCGGAGGTGAGCTAGAAA-3’
actin F	NM_001101	5’-ATCATGTTTGAGACCTTCAA-3’
actin R		5’-CATCTCTTGCTCGAAGTCCA-3’
